# Remote Analysis of Respiratory Sounds in Patients With COVID-19: Development of Fast Fourier Transform–Based Computer-Assisted Diagnostic Methods

**DOI:** 10.2196/31200

**Published:** 2022-07-19

**Authors:** Gregory Furman, Evgeny Furman, Artem Charushin, Ekaterina Eirikh, Sergey Malinin, Valery Sheludko, Vladimir Sokolovsky, David Shtivelman

**Affiliations:** 1 Physics Department Ben-Gurion University of the Negev Beer-Sheva Israel; 2 Department of Pediatric EA Vagner Perm State Medical University Perm Russian Federation; 3 Department of Ear, Nose and Throat EA Vagner Perm State Medical University Perm Russian Federation; 4 Central Research Laboratory EA Vagner Perm State Medical University Perm Russian Federation; 5 Perm Regional Clinical Infectious Diseases Hospital Perm Russian Federation

**Keywords:** COVID-19, audio analysis, remote computer diagnosis, respiratory sounds, respiratory analysis, modeling, computer-assisted methods, diagnostics

## Abstract

**Background:**

Respiratory sounds have been recognized as a possible indicator of behavior and health. Computer analysis of these sounds can indicate characteristic sound changes caused by COVID-19 and can be used for diagnostics of this illness.

**Objective:**

The aim of the study is to develop 2 fast, remote computer-assisted diagnostic methods for specific acoustic phenomena associated with COVID-19 based on analysis of respiratory sounds.

**Methods:**

Fast Fourier transform (FFT) was applied for computer analysis of respiratory sound recordings produced by hospital doctors near the mouths of 14 patients with COVID-19 (aged 18-80 years) and 17 healthy volunteers (aged 5-48 years). Recordings for 30 patients and 26 healthy persons (aged 11-67 years, 34, 60%, women), who agreed to be tested at home, were made by the individuals themselves using a mobile telephone; the records were passed for analysis using WhatsApp. For hospitalized patients, the illness was diagnosed using a set of medical methods; for outpatients, polymerase chain reaction (PCR) was used. The sampling rate of the recordings was from 44 to 96 kHz. Unlike usual computer-assisted diagnostic methods for illnesses based on respiratory sound analysis, we proposed to test the high-frequency part of the FFT spectrum (2000-6000 Hz).

**Results:**

Comparing the FFT spectra of the respiratory sounds of patients and volunteers, we developed 2 computer-assisted methods of COVID-19 diagnostics and determined numerical healthy-ill criteria. These criteria were independent of gender and age of the tested person.

**Conclusions:**

The 2 proposed computer-assisted diagnostic methods, based on the analysis of the respiratory sound FFT spectra of patients and volunteers, allow one to automatically diagnose specific acoustic phenomena associated with COVID-19 with sufficiently high diagnostic values. These methods can be applied to develop noninvasive screening self-testing kits for COVID-19.

## Introduction

The World Health Organization (WHO) reported that till October 16, 2021, about 241 million people were infected with the novel coronavirus (COVID-19) worldwide and about 18 million people currently have the disease [1]. СOVID-19 is a public health problem in all countries regardless of their level of development.

It is known that SARS-CoV-2 causes a severe lower respiratory disease with high mortality and evidence of systemic spread [2]. The virus is able to actively multiply in the epithelium of the airways. Intense cough is 1 of the main symptoms of COVID-19. It is known that the highest density of cough receptors is in the larynx [3]. Anatomically, a dry cough can be associated with the effect of the virus on the cough receptors of the larynx due to infection with COVID-19. SARS-CoV-2 can penetrate into the smallest airways, where it infects cells and causes bilateral pneumonia, often with respiratory failure [4-7]. The damage of various airways, caused by SARS-CoV-2, alters sound formation in the patient and changes the characteristics of respiratory sounds. Detection of characteristic respiratory sounds (cough, wheezes, asthma wheezing, shortness of breath, etc) is a widely used way of diagnostics of pulmonary diseases, which is applied to develop new computer-assisted diagnostic methods (eg, [8-12] and references therein).

At present, diagnostics of COVID-19 is based on clinical symptoms, chest X-ray/computed tomography (CT), and coronavirus tests (polymerase chain reaction [PCR]), molecular tests, antigen tests, and specific antibodies to SARS-CoV-2) [6,7,13]. New and more contagious COVID-19 virus strains are appearing in the United Kingdom, the Republic of South Africa, Vietnam, and some other countries; in India, the daily number of new infected persons was up to 300,000 in May 2021. Therefore, it is desirable to develop vast, cheap, and widely available remote methods of COVID-19 diagnostics. One of these methods can be based on computer-assisted analysis of respiratory sounds of the patient and on comparison of the sound characteristics between a patient and a healthy volunteer.

The objectivity of auscultatory diagnostics can be significantly enhanced by using digitized audio signals and computer processing of these signals. Automated adventitious sound detection or classification is a promising solution to overcome the limitations of conventional auscultation and to assist in the monitoring of relevant diseases, such as asthma, chronic obstructive pulmonary disease (COPD), and pneumonia [14]. Olvera-Montes et al [15] used the detection of respiratory crackle sounds through an Android smartphone–based system for the diagnostics of pneumonia and monitoring of the patient’s state.

Reyes et al [16] used a smartphone-based system for automated bedside detection of crackle sounds in patients with diffuse interstitial pneumonia. The performance of automated detection was analyzed using (1) synthetic fine and coarse crackle sounds randomly inserted into basal respiratory sounds acquired from healthy subjects with different signal-to-noise ratios and (2) real bedside-acquired respiratory sounds from patients with interstitial diffuse pneumonia. In simulated scenarios, for fine crackles, an accuracy ranging from 84.86% to 89.16%, a sensitivity ranging from 93.45% to 97.65%, and a specificity ranging from 99.82% to 99.84% were found. The detection of coarse crackles was found to be a more challenging task in the simulated scenarios. In the case of real data, the results show the feasibility of using the developed mobile health system in a clinical noncontrolled environment to help the expert in evaluating the pulmonary state of a subject.

The overview concerns the potential for computer audition (CA), that is, the use of speech and sound analysis by artificial intelligence to help in COVID-19 diagnostics [17]. Automatic recognition and monitoring of breathing, dry and wet coughing or sneezing sounds, speech under cold, eating behavior, sleepiness, or pain are used. Schuller et al [17] concluded that CA appears to be ready for implementation of (pre-) diagnosis and monitoring tools.

It was considered that the acquired breathing sounds can be analyzed using advanced signal processing and in tandem with new deep machine learning and pattern recognition techniques to separate the breathing phases, estimate the lung volume, estimate oxygenation, and further classify the breathing data input into healthy or unhealthy cases [18]. Computer analysis of breath sounds can be important for identification of specific changes in these sounds, caused by COVID-19.

Brown et al [19] used the exploring automatic diagnostics of COVID-19 from crowdsourced respiratory sound data. The results of early works [16-19] and references therein allow one to suggest that respiratory sounds can be useful in COVID-19 diagnostics.

The purpose of this study is to 2 develop fast, remote methods of diagnostics of specific acoustic phenomena associated with COVID-19 based on computer-assisted analysis of respiratory sounds. The developed methods are based on analysis of fast Fourier transform (FFT) spectra of respiratory sounds recorded near the mouth. We proposed to use a personal computer, a modern mobile telephone, or a smartphone for registration, recording of respiratory sounds, and their analysis. The developed methods can be applied as additional screening methods of COVID-19 diagnostics and as personalized screening self-testing kits for COVID-19. Such self-tests would serve as an early step before further procedures ordered by a doctor (PCR test, lung CT, X-ray, etc). We restricted our work to consideration of breathing sounds (and not cough and voice samples). Lung diseases, such as asthma, COPD, and pneumonia, cause specific changes in the FFT spectra of respiratory sounds in the frequency range from 100 to 2500 Hz, and this range is usually considered during development of computer-assisted diagnostic methods (eg, [8-12,20-22] and references therein). We considered the larger frequency range up to 6000 Hz, and it was shown that for diagnostics of COVID-19, the frequency range from 2000 to 6000 Hz is significant. This allows us to diagnose specific acoustic phenomena associated with COVID-19 for patients with other lung diseases as well.

## Methods

### Patients

In this study, 14 patients with COVID-19 and 17 healthy volunteers participated. COVID-19 in the patients was diagnosed using medical methods, such as analysis of clinical symptoms, chest X-ray and CT, coronavirus tests (PCR test for SARS-CoV-2 RNA [the main method], specific antibody test for SARS-CoV-2). The clinical examination of the patients and the recording of their respiratory sounds were carried out at Perm Infectious Hospital, and the volunteers’ respiratory sounds were recorded at Perm State Medical University (Perm, Russia).

### Ethical Considerations

This study complied with the Declaration of Helsinki (adopted in June 1964, Helsinki, Finland), revised in October 2000 (Edinburg, Scotland) and was overseen by the independent ethics committee of Perm State Medical University (approval code: 5/21). Written agreements from the patients and volunteers were obtained.

### Methods of Recording Respiratory Sounds

Doctors performed all the recordings, and patients and volunteers were instructed to remain calm and to breathe easily. No special measures to reduce ambient noise were applied. The respiratory sounds of patients were recorded in m4a format using a Honor dua-1 22 smartphone at a distance of 2 cm from the mouth for about 20 s; the sampling rate was 48 kHz. The respiratory sounds of volunteers were recorded near the mouth using a mobile telephone (the sampling rate was 44.1 kHz, mp3 format) and a computer-based recording system (the sampling rate was 96 kHz, wav format) [20-25]. Our comparison of the records carried out with the help of various devices showed that although the mp3 and m4a formats compress a signal, the FFT spectra of a sound recorded in the wav, mp3, and m4a formats are practically the same. To analyze respiratory sounds, we proposed to consider the normalized FFT spectra that largely decrease the influence of the recording format.

Simultaneous recordings of respiratory sounds in the same point of a patient in different formats were made. The normalized integral characteristics of the FFT spectra used for diagnostics differed by less than 3% for different formats.

The beginnings and ends of the recordings made using the smartphone and the mobile telephone contained temporal parts, in which respiratory sounds are not recorded. In these parts, there are short impulses, the amplitudes of which can be much larger than the respiratory sound maximum. The impulses do not reflect the processes in the airways. The applied preliminary processing removed these parts.

### Diagnostic Methods

The 2 proposed diagnostic methods were based on the fact that lung diseases cause changes in the airways and these changes are reflected in the spectra of respiratory sounds. This approach has been applied in the development of computer-assisted methods of diagnostics of various lung diseases [8-12,20-26]. These developed methods were based on the analysis of the FFT spectra of respiratory sounds in the frequency range from 100 to 2500 Hz and the comparison of the spectra of patients and healthy volunteers.

**Figure 1:**
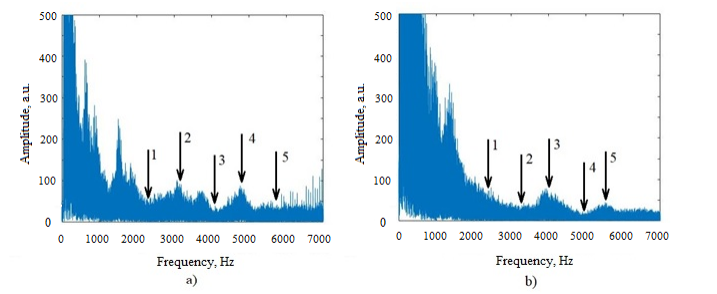
Amplitudes of FFT harmonics for the first healthy volunteer (a) and the first patient with COVID-19 (b). The amplitudes are given in arbitrary units. FFT: fast Fourier transform.

We proposed to compare different parts of the FFT spectrum of a patient with COVID-19 in a higher frequency range from 2000 to 6000 Hz. Examples of the amplitude-frequency dependences (spectra) of FFT for a patient with COVID-19 and a healthy volunteer are presented in Figure 1. In many cases, the FFT spectra of volunteers do not possess well-defined maximums and minimums, as shown in Figure 1a. One can see several differences (Table 1) in the spectra for a volunteer and a patient: the maxima and minima of their spectra are located in various frequency ranges; these differences could be used to formulate potential healthy-ill criteria, which are presented in Table 2. Similar locations of the maxima and minima were observed, for example, in the spectra for the first, third, and ninth patients. The clearly seen amplitude increase in the frequency range from 1000 to 1500 Hz in the spectrum for the first patient (Figure 1b) can be a result of concomitant disease (upper respiratory tract infection).

In Table 2:




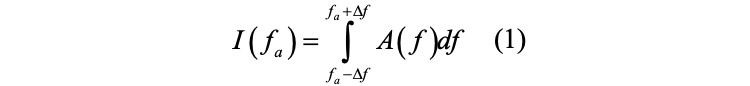




where f_a_ is the frequency of the extremum, A(f) is the harmonic amplitude at frequency f, and Δf is the half of the frequency range, chosen equal to 300 Hz. In the program, the integral is replaced by a sum of harmonic amplitudes with a frequency in the range from (f_a_ – Δf) to (f_a_ + Δf).

The criteria under test were formulated as ratios of the integrals of the harmonic amplitudes over various frequency ranges (Table 2 and Equation 1). So, the criteria values were independent of the breathing intensity.

Adventitious sounds caused by an illness change not only the amplitude-frequency dependence of the respiratory sound FFT spectrum but also the frequency-amplitude dependence. The second proposed method is based on analysis of differences between the frequency-amplitude dependences of FFT spectra for patients and volunteers.

The moments of the frequency (MF) distribution can be considered as potential healthy-ill criteria:




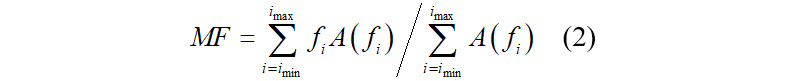




and




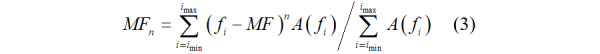




where i_min_ and i_max_ are the harmonic numbers corresponding to the minimal (f_min_=2000 Hz) and maximal (f_max_=5900 Hz) frequencies, respectively, and f_i_ is the frequency of the i-th harmonic.

The MF distribution was also independent of the breathing intensity.

**Table 1:** Comparison of the FFT^a^ spectra of a volunteer and a patient with COVID-19.

Volunteer	Patient
Minimum at about 2300 Hz	No extremum in the frequency range
Maximum at 3100 Hz	Minimum at 3300 Hz
Minimum at 4100 Hz	Maximum at 3900 Hz
Maximum at 4900 Hz	Minimum at 5000 Hz
No extremum at a frequency above 5300 Hz	Maximum at 5600 Hz

^a^FFT: fast Fourier transform.

**Table 2:** Healthy-ill criteria.

Criteria	Healthy	Ill
k_1_ = I(2300)/I(3200)	k_1_<1	k_1_>1
k_2_ = I(3200)/I(4000)	k_2_>1	k_2_<1
k_3_ = I(4000)/I(5000)	k_3_<1	k_3_>1
k_4_ = I(5000)/I(5600)	k_4_>1	k_4_<1

## Results

### The First Method

The results of the calculation of potential healthy-ill criteria k_1_, k_2_, k_3_, and k_4_ are presented in Tables 3 and 4.

**Figure 2:**
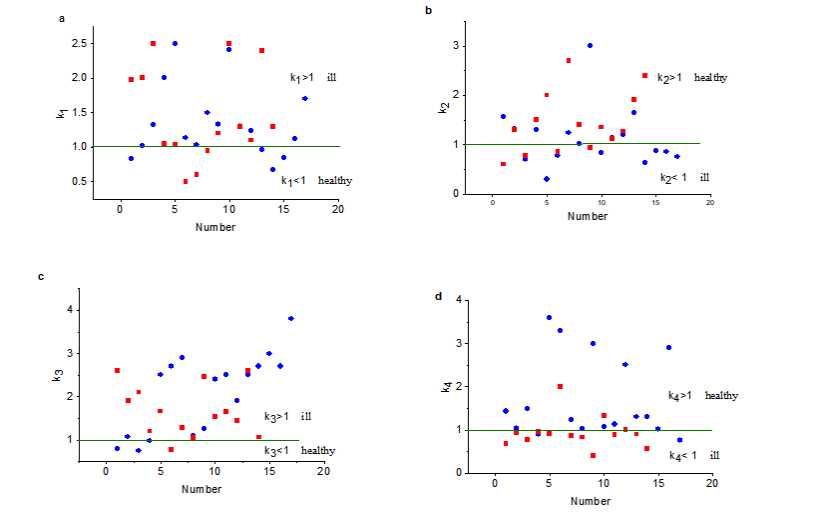
Healthy-ill criteria according to Tables 3 and 4: blue circles for volunteers and red squares for patients.

For the reader’s convenience, Figure 2 presents the distribution of the criteria through patients with COVID-19 and volunteers. Comparing the results presented in Tables 3 and 4 and Figure 2, one can see that the most reliable result is given by the high-frequency criterion k_4_.

For healthy volunteers, the criterion should be >1; this was correct in T_N_=15 (88.2%) of 17 cases and incorrect in F_N_=2 (11.8%) cases. For patients with COVID-19, the criterion should be <1; this was observed in T_P_=11 (78.6%) of 14 cases. COVID-19 was not diagnosed in F_P_=3 (21.4%) cases.

**Figure 3:**
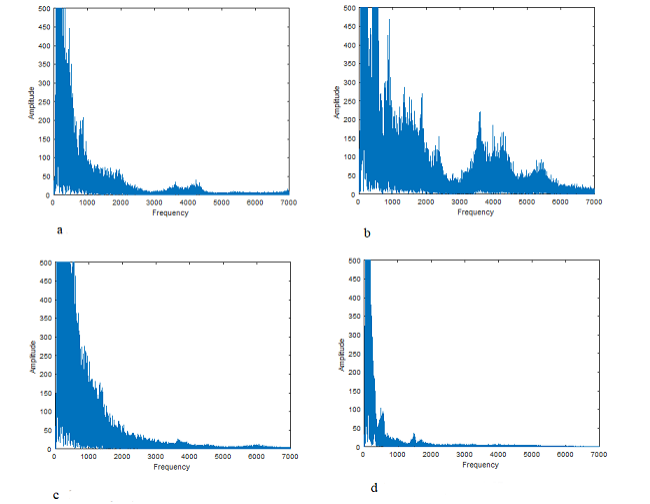
Change in the respiratory sound FFT spectrum of a patient with COVID-19 in the disease process. On the first day, the illness was diagnosed using the PCR test; on the last (11th) day, the person was diagnosed as healthy. The amplitudes are given in arbitrary units. FFT: fast Fourier transform; PCR: polymerase chain reaction.

The criterion for the 12^th^ patient was close to the boundary value of 1. The second proposed method also had the healthy-ill criterion close to the boundary value (Figure 3). Analysis of the recording for the patient showed that the outside noise was high, and this can cause incorrect diagnostics.

Additionally, we applied the proposed method for diagnostics of COVID-19 using the respiratory sound recordings of 30 patients and 26 healthy persons (ages 11-67 years, 34, 60%, women). None of them reported that they had other lung diseases. These recordings were made by the persons themselves, who agreed to be tested at home, using a mobile telephone. The sound recordings were passed to us through WhatsApp. The cases of COVID-19 illness were confirmed by PCR tests, and in all cases, the illness was asymptomatic or mild. The proposed method gave the correct diagnosis for 26 (86.7%) of 30 patients and 22 (84.6%) of 26 healthy ones. The PCR tests for persons who were incorrectly diagnosed as ill gave negative results.

Several patients made a few recordings during the process of the disease. Development of a spectrum of a patient is presented in Figure 3. Though the spectrum varied noticeably, the k_4_ criterion was less than 1 during the illness. The pathological process was characterized by a clearly seen increase in harmonic amplitudes in a wide frequency range. The third-day spectrum corresponded to the most severe condition of the person (according to their message).

**Table 3:** Criteria k_1_, k_2_, k_3_, and k_4_ for patients with COVID-19.

Patient number	Age (years), gender (F=female, M=male)	Diagnosis	k_1_	k_2_	k_3_	k_4_
1	18, F	COVID-19, upper respiratory tract infection	>1	<1	>1	<1
2	80, F	COVID-19, unilateral pneumonia	>1	>1	>1	<1
3	47, F	COVID-19, bilateral pneumonia	>1	<1	>1	<1
4	58, F	COVID-19, bilateral pneumonia	>1	>1	>1	<1
5	62, M	COVID-19, pneumonia	>1	>1	>1	<1
6	65, F	COVID-19, pneumonia	<1	<1	<1	>1
7	28, F	COVID-19, unilateral pneumonia with hydrothorax, HIV infection	<1	>1	>1	<1
8	75, M	COVID-19, upper respiratory tract infection	<1	>1	>1	<1
9	38, F	COVID-19, upper respiratory tract infection, exacerbation of COPD^a^	>1	<1	>1	<1
10	36, F	COVID-19, pneumonia	>1	>1	>1	>1
11	20, M	COVID-19, pneumonia	>1	>1	>1	<1
12	56, M	COVID-19, pneumonia	>1	>1	>1	~1
13	20, M	COVID-19, pneumonia	>1	>1	>1	<1
14	32, M	COVID-19, pneumonia	>1	>1	>1	<1

^a^COPD: chronic obstructive pulmonary disease.

**Table 4:** Criteria k_1_, k_2_, k_3_, and k_4_ for volunteers.

Volunteer number	Age (years), gender (F=female, M=male)	k_1_	k_2_	k_3_	k_4_
1	22, F	<1	>1	<1	>1
2	47, M	>1	>1	>1	>1
3	48, F	>1	<1	<1	>1
4	17, M	>1	>1	<1	<1
5	8, M	>1	<1	>1	>1
6	5, F	>1	<1	>1	>1
7	5, F	>1	>1	>1	>1
8	11, M	>1	>1	>1	>1
9	5, M	>1	>1	>1	>1
10	14, M	>1	<1	>1	>1
11	5, F	>1	>1	>1	>1
12	12, F	>1	>1	>1	>1
13	9, M	<1	>1	>1	>1
14	10, F	<1	<1	>1	>1
15	10, F	<1	<1	>1	>1
16	8, M	>1	<1	>1	>1
17	14, M	>1	<1	>1	<1

### The Second Method

**Figure 4:**
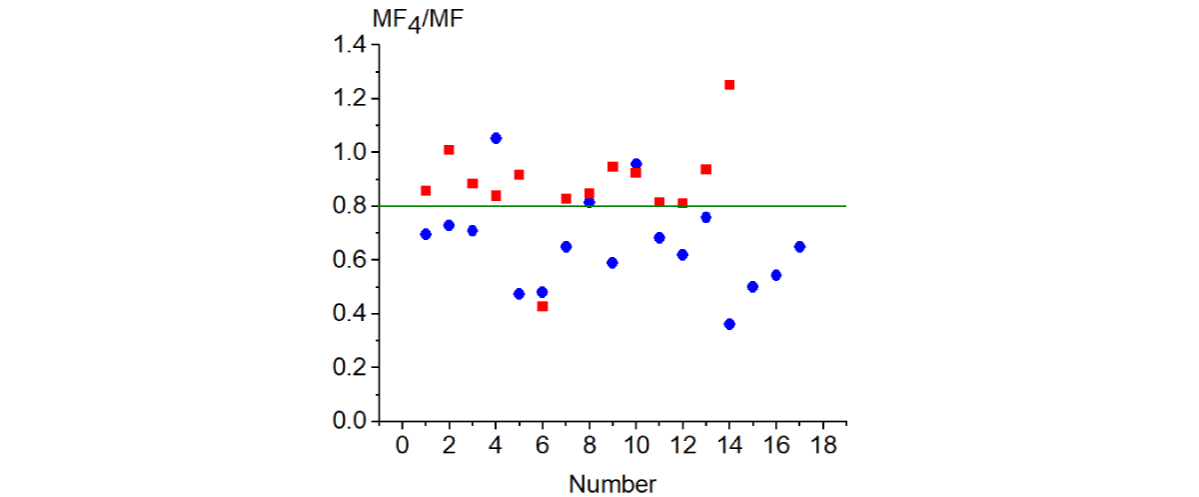
Healthy-ill criterion (MF_4_/MF)×10^−9^: blue circles for volunteers and red squares for patients. Each number on the horizontal axis indicates the number of a patient or a volunteer according to Tables 3 and 4, respectively. The blue line corresponds to the boundary value of 0.8. MF: moments of the frequency.

We compared the values of MF and MF_n_ (n=2, 3, and 4) as well as MF_n_/MF and MF_n_/MF_2_. The best result was obtained for MF_4_/MF (Figure 4). In this figure, the blue line corresponds to the boundary value, which was selected by us as equal to 0.8: If (MF_4_/MF)×10^−9^ is >0.8, the examined person is ill; if it is <0.8, the person is healthy. The second method, like the first one, gave incorrect diagnostics for the sixth patient and overdiagnosis for the fourth volunteer. The respiratory sound recording for this volunteer was characterized by a relatively low level of a signal and high noise. The overdiagnosis for the fourth volunteer could be the result of low quality of the recorded signal.

The second method correctly diagnosed patients as sick in T_P_=13 (92.8%) of 14 cases and volunteers as healthy in T_N_=14 (82.3%) of 17 cases. The method misdiagnosed patients as healthy only once, F_P_=1 (7.2%), and healthy volunteers as sick in F_N_=3 (17.7%) cases.

The second method applied to the respiratory sounds recorded by persons at home demonstrated a diagnostics effectivity that was close to that of the first method.

The results allow us to assess the main characteristics of the 2 proposed methods. Here, for characterization of the proposed methods, we used sensitivity, specificity, and the Youden index. The sensitivity of a method is determined by the formula [27]




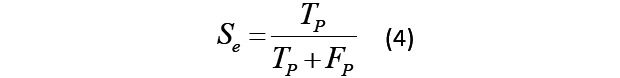




and its specificity as




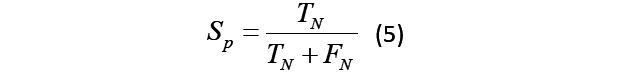




The sensitivity and specificity of the first method from Tables 1 and 2 were estimated as S_e_=0.786 and S_p_=0.882, respectively, and for the second method as (Figure 2) as S_e_=0.93 and S_p_=0.824. Here, we considered the results obtained only for people who were tested at the hospital.

The Youden index is calculated using the following formula:




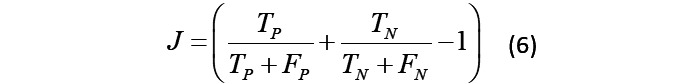




The Youden index for the first method was about 0.67 and for the second was 0.754.

For the reader’s convenience, these results and the sensitivity, specificity, and Youden index for both methods are presented in Table 5.

**Table 5:** Comparison of the 2 proposed methods.

Method	T_P_	F_P_	T_N_	F_N_	S_e_	S_p_	J
First	11	3	15	2	0.786	0.882	0.67
Second	13	1	14	3	0.93	0.824	0.754

## Discussion

### Principal Findings

The proposed computer-assisted diagnostic methods for COVID-19, which are based on analysis of respiratory sounds recorded near the mouth, demonstrated high diagnostic accuracy. For people tested at the hospital, the second method demonstrated better characteristics (sensitivity of 0.93, specificity of 0.824, and Youden index of 0.754) than the first one.

Both methods demonstrated close diagnostic characteristics when analyzing respiratory sound recordings made by persons themselves using a mobile telephone at home and submitted to us through WhatsApp. The proposed methods correctly diagnosed 86.7% of patients and 84.6% of healthy ones. These results demonstrate the possible application of the proposed methods for remote diagnostics.

Although a relatively low number (due to pandemic limitations) of the examined patients with COVID-19 and healthy volunteers did not allow estimating the method characteristics with high accuracy, the proposed methods correctly diagnose patients with COVID-19 in a wide age range, and the proposed criteria of healthy/ill are independent of the patient’s age, sex, etc, as well as concomitant diseases, such as upper respiratory tract infection, pneumonia, exacerbation of COPD, and bilateral pneumonia (Table 3).

The patient and volunteer groups contained members of various genders and ages (from 5 to 80 years).

High diagnostic characteristics of the proposed methods independent of age were achieved due to comparison of various parts of the respiratory sound FFT spectrum. For example, in the first method, the ratio k_4_ of integrals over various frequency ranges was determined and compared with the boundary value: If k_4_>1, the examined person is healthy, and if k_4_<1, the person is ill. The ratio is independent of the recording devices and sampling rate, and as our results showed, the boundary value does not depend on the individual patient characteristics and even on concomitant diseases (Table 3).

### Comparison With Prior Work

**Figure 5:**
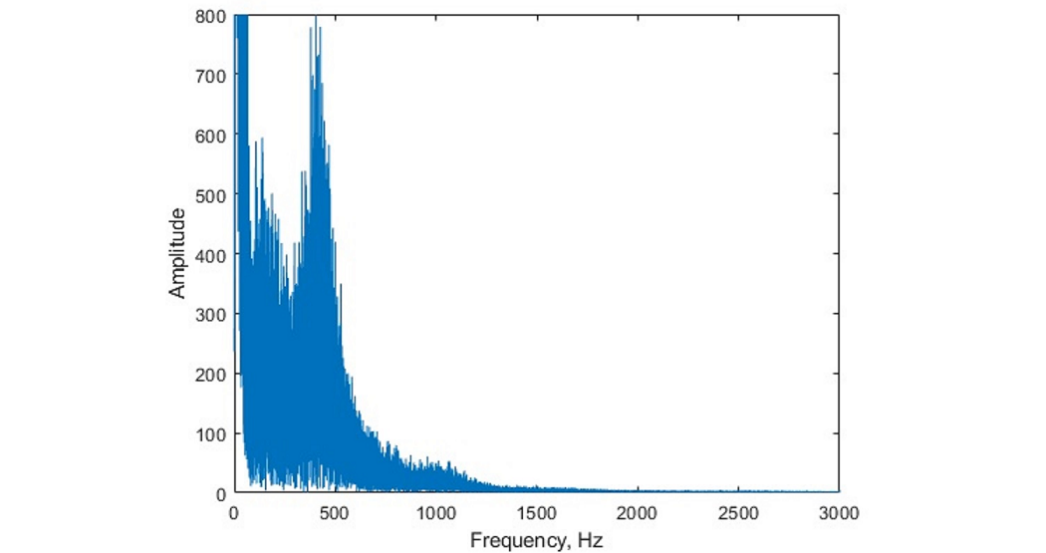
FFT spectra of sound signals for an asthmatic patient. The amplitudes are given in arbitrary units. FFT: fast Fourier transform.

Each lung disease is characterized by specific changes in the airways or lungs. These changes cause abnormal (adventitious) sounds, which can be separated into several types (wheezing, stridor, crackles, etc). These abnormal sounds are characterized by their durations and specific frequency ranges being below 2500 Hz [8,10,12]. For example, bronchial asthma is characterized by airway obstruction and inflammatory process, which covers all airways, from the central to the peripheral parts of the tracheobronchial tree (small bronchi) [21]. Asthmatic changes in the lungs cause typical respiratory sounds, with the main frequency in low-frequency ranges between 100 and 1000 Hz [12,28,29] and between 400 and 1600 Hz [12,30] (Figure 5). For analysis of respiratory sounds of patients with pulmonary diseases (asthma, pneumonia, HIV infection, etc) and development of computer-assisted diagnostic methods, the frequency range from 100 to 2500 Hz is usually considered.

For diagnostics of specific acoustic phenomena associated with COVID-19, we proposed to consider the high-frequency range of respiratory sound FFT spectra. It can be assumed that аn upper respiratory tract injury leads to the appearance of changes in a higher-frequency part of the spectrum; it can be associated with COVID-19 [31]. Severe forms of tracheobronchitis were consistently present in 88% of COVID-19 cases [32-34]. This assumption is confirmed by our results: the most reliable criterion is the k_4_ criterion, which is determined for the high-frequency range of the FFT spectrum, from 4700 to 5900 Hz (the first proposed method).

A decrease in the diagnostic value of the k_1_, k_2_, and k_3_ criteria can be the result of concomitant diseases, which cause changes in the respiratory sounds in the lower-frequency range [8,10,12]. Another reason of the low accuracy of diagnostics based on the k_1_, k_2_, and k_3_ criteria can be a higher sensitivity of the parameters f_a_ and Δf at low frequencies to individual characteristics of patients (age, sex, weight, etc) and also to fatigue and anxiety.

The second proposed method of computer-assisted diagnostics of COVID-19 is also based on the consideration of the high-frequency range of the FFT spectrum, from 2000 to 6000 Hz.

The high diagnostic accuracy is achieved in both methods due to our offer to compare various parts of the FFT spectrum of a patient (volunteer). This allows us to minimize the influence of the breathing intensity as well as the gender and age dependences of the FFT spectrum.

One of the ways to increase the diagnostic values of the proposed computer-assisted methods is to create a big database and determine the parameters (f_a_ and Δf for the first method and f_min_ and f_max_ for the second one) using machine learning.

The proposed methods for COVID-19 diagnostics are based on the consideration of the high-frequency ranges of FFT spectra. The most reliable result is given by the high-frequency criterion k_4_ for the frequency range above 4700 Hz. Other lung illnesses do not cause abnormal respiratory sounds (adventitious sounds) in the considered frequency range; changes caused by them are between 50 and 2500 Hz (see Figure 5 and [8,10,12,28,35]). This fact and the independence of the proposed criteria of the concomitant diseases allow us to assume that the criteria can be used for diagnostics of COVID-19. We analyzed FFT spectra for several patients with other lung diseases (without COVID-19), such as asthma (Figure 5), bilateral pneumonia, pneumonia, and upper respiratory tract infection, and did not find these specific changes in the high-frequency range above 4700 Hz.

### Limitations

The proposed screening self-tests would serve as a preliminary step before further procedures are ordered by a doctor. The results of the screening self-tests should be confirmed by other diagnostic methods (chest X-ray/CT and coronavirus tests, such as PCR test, antigen test, and specific SARS-CoV-2 antibody test).

### Conclusion

The high-frequency range of the respiratory sound FFT spectrum contains information about the health state of the examined person. The proposed computer-assisted methods based on analysis of this spectrum part can be applied as fast, remote additional screening methods (telemedicine) for specific acoustic phenomena associated with COVID-19. The methods demonstrate sufficiently high diagnostic values. The methods can be a basis for the development of noninvasive screening self-testing kits for COVID-19. To increase the accuracy and reliability of the methods, a big database of respiratory sounds of patients with COVID-19 and volunteers should be created.

## Abbreviations

CA: computer audition
COPD: chronic obstructive pulmonary disease
CT: computed tomography
FFT: fast Fourier transform
MF: moments of the frequency
PCR: polymerase chain reaction
